# Supplementary material for: Low-Carbohydrate Diet Inhibits Different Advanced Glycation End Products in Kidney Depending on Lipid Composition but Causes Adverse Morphological Changes in a Non-Obese Model Mice
Source: Nutrients. 2019 Nov 16;11(11):2801. doi: 10.3390/nu11112801 (PMC6893679; doi:10.3390/nu11112801)
Supplement: Supplementary file 1 [file nutrients-11-02801-s001.pdf]

Table S1. Composition of the experimental diets. Control (control diet); LC (low-carbohydrate diet); MCT-LC (LC diet containing medium-chain triglyceride).

	**Control**	**LC**	**MCT-LC**
PFC energy ratio (%)			
Carbohydrate	64	20	20
Protein	20	35	35
Fat	16	45	45
Energy (kcal/g)	4.0	4.0	4.0
Ingredient(g/kg)			
Casein	200.0	345.0	345.0
Cornstarch	397.5	0	0
Maltodextrin 10	132.0	132.0	132.0
Sucrose	100.0	64.9	64.9
cellulose	50.0	50.0	50.0
Soybean oil	70.0	70.0	70.0
Lard	0.0	130.0	0
MCT oil	0.0	0.0	130.0
Mineral mix S10022G	35.0	0.0	0.0
Mineral mix S10022C	0.0	3.5	3.5
Calcium carbonate	0.0	12.5	12.5
Potassium citrate	0.0	6.5	6.5
Potassium phosphate	0.0	1.8	1.8
Sodium chloride	0.0	2.6	2.6
Vitamin mix V10037	10.0	10.0	10.0
L-Cystine	3.0	5.2	5.2
Choline bitartrate	2.5	2.5	2.5
t-Butylthdroquinone	0.014	0.014	0.014

Control, control diet; LC, low-carbohydrate diet high in lard; MCT-LC, low-carbohydrate diet high in medium-chain triglyceride.
